# The role of genetic testing in suspected fulminant myocarditis: A case report

**DOI:** 10.1016/j.ymgmr.2023.101000

**Published:** 2023-08-22

**Authors:** Raffaella Mistrulli, Caterina Micolonghi, Federico Follesa, Marco Fabiani, Erika Pagannone, Giulia D'Amati, Carla Giordano, Silvia Caroselli, Camilla Savio, Aldo Germani, Antonio Pizzuti, Vincenzo Visco, Simona Petrucci, Speranza Rubattu, Maria Piane, Camillo Autore

**Affiliations:** a Department of Clinical and Molecular Medicine, Faculty of Medicine and Psychology, Sapienza University of Rome, 00189 Rome, Italy; b Department of Experimental Medicine, Faculty of Medicine and Dentistry, Sapienza University of Rome, 00161 Rome, Italy; c ALTAMEDICA, Human Genetics, 00198 Rome, Italy; d Department of Radiological, Oncological and Pathological Sciences, Sapienza, University of Rome, Rome, Italy; e Reproductive Genetics, Juno Genetics, 00188 Rome, Italy; f S. Andrea University Hospital, 00189 Rome, Italy; g Medical Genetics Unit, IRCCS Mendel Casa Sollievo della Sofferenza, 71013 San Giovanni Rotondo, Italy; h IRCCS Neuromed, Pozzilli, IS 86077, Italy; i San Raffaele Cassino, 03043, Italy

**Keywords:** Arrhythmogenic cardiomyopathy, Acute myocarditis, Biopsy, Genetics, *PKP2*

## Abstract

ACM is a rare hereditary heart disease characterized by a progressive fibro-fatty replacement of the myocardium that can affect either the right or the left ventricle or both. It is mainly caused by variants in the desmosome genes with autosomal dominant transmission and incomplete penetrance. The disease shows a wide spectrum of clinical manifestations, including ventricular arrhythmias, HF and myocarditis. The latter is considered a ‘hot phase’ in the natural history of the disease and must therefore be distinguished from the isolated AM, which is frequently due to viral infections. Our case report is an example of how an AM, as the first manifestation of the disease, helped to reach a diagnosis of ACM through the genetic analysis. In fact, the multi-parametric investigation, which also included CMR and EMB, revealed controversial aspects that led us to perform the genetic test. The latter revealed a heterozygous pathogenic variant in the *PKP2* that was considered definitive proof of ACM.

## 1 Introduction

Most cases of AM, an inflammatory condition of the myocardium, are caused by a viral infection and/or an autoimmune reaction. Although two-thirds of cases recover spontaneously, the remaining patients develop LV dysfunction and arrhythmias. Based on post-mortem studies, AM explains 3% to 12% of all cases of SCD [1]. Recent studies have demonstrated a genetic predisposition in a subset of AM, which may herald the onset of either DCM or ACM. However, the link between these conditions and the frequency of the association remain still unclear [2,3]. ACM is a rare inherited cardiac disease characterized by a progressive fibro-fatty replacement of the myocardium. It is caused mostly by variants in desmosome genes with autosomal dominant transmission and incomplete penetrance [3,4]. To date, according to Clinical Genome Resource (ClinGen, https://clinicalgenome.org/), 17 genes are classified as having definitive evidence for ACM: *PKP2, DSG2, DES, DSP*, *JUP*, *DSC2*, *PLN, CTNNA3, MYL3, TTN, SCN5A, TJP1, LMNA, CDH2, MYBPC3, TGFB3* and *MYH7* [5]. The clinical diagnosis of ACM is often challenging due to the broad spectrum of phenotypes which includes arrhythmias, SCD and/or HF and the lack of a family history in approximately 50% of patients. Because of the severity of symptoms, the early identification of carriers is fundamental. Some studies focused on the identification of ECG abnormalities that may indicate the presence of an underlying cardiomyopathy, but more studies are required to identify signs of an early phase of the disease [6].

Interestingly, AM episodes have been described in patients with ACM suggesting that inflammation may play a part in the pathogenesis of the disease. Chest discomfort and elevated troponin level represent the symptom and sign, respectively, of the *“*hot phases” of ACM which mimic a viral AM during disease progression [7]. Furthermore, AM may act as an environmental modifier in healthy patients with a genetic predisposition to inherited cardiomyopathies, causing a phenotypic expression of the disease [8]. In this report, we describe a case of AM where the genetic test revealed a heterozygous pathogenic variant in the *PKP2*.

## 2 Case presentation

A 65-year-old female with no family history of cardiomyopathy, with known hypertension and dyslipidemia presented to our hospital with persistent moderate-intensity chest pain and dyspnoea. Three weeks earlier she had an episode of high fever lasting a couple of days, which had resolved spontaneously, followed by the onset of mild-to-moderate dyspnoea. At admission, the patient was tachypnoeic and still symptomatic although apyretic. Blood pressure was 150/100 mmHg and SpO2 was 98%. Cardiovascular examination was unremarkable and respiratory examination revealed bi-basal crepitations without peripheral oedema and jugular vein distension. The initial ECG showed sinus tachycardia, low voltages, and mild ST-elevation in the infero-lateral leads. The ECHO showed akinesia of the apical and periapical segments with a severe reduction of LVEF (25%) and moderate mitral regurgitation. There was no pericardial effusion. The high-sensitivity troponin test detected significantly elevated troponin level.

The patient was diagnosed with ST elevation myocardial infarction and was urgently transported to the catheterization laboratory. The coronary angiography showed no stenosis of the epicardial vessel, and the patient was subsequently transferred to the ICU.

At ICU admission, diuretic, nitro-glycerine, and inotropic therapy with dobutamine was initially administered. Dobutamine was subsequently stopped due to the appearance of PVC and NSVT. Nitrates were also discontinued due to the appearance of hypotension. Pharmacological therapy was optimised by starting a cycle of levosimendan with vasopressor support by noradrenaline. In addition, the patient had febrile rises for which empiric broad-spectrum antibiotic therapy was administered. Troponin I continued to rise and remained critically elevated (700,000 pg/mL) for several days. The remaining blood tests showed neutrophilic leukocytosis and elevated PCR (5.7 mg/L). B-type natriuretic peptide level was 1511 pg/mL. The subsequent ECHO confirmed severely depressed LVEF (25%), with akinesia of the apex and para-apical segments and showed increased parietal thickness as from intramyocardial oedema. A minimal pericardial effusion was also detected.

**Fig. 1:**
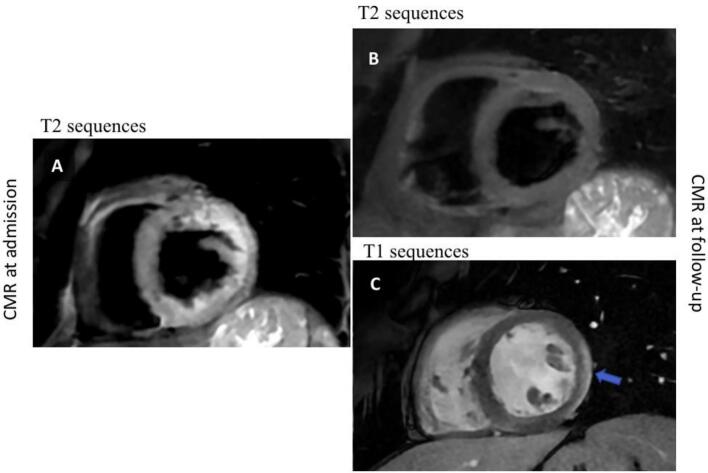
Cardiac magnetic resonance imaging studies. A. CMR of the patient at admission, showing an extensive oedema at T2 sequences at the mid and apical segments of the lateral and inferior walls. The initial LVEF was 25%. B-C. Follow-up CMR showing the absence of oedema in the T2 sequences (B) and a multifocal subepicardial LGE within the lateral wall, as indicated by the arrow (C).

**Fig. 2:**
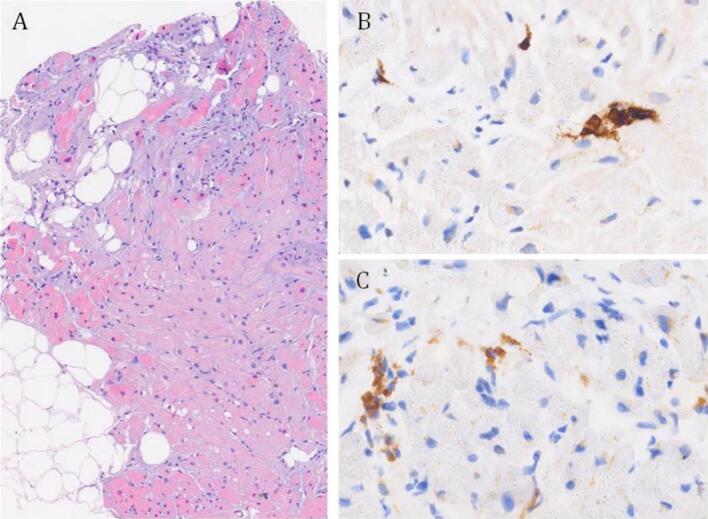
A. EMB showing an increased interstitial cellularity and areas of fibro-adipose myocardial replacement (hematoxylin-eosin, original magnification 10X). B. A small cluster of T lymphocytes (CD3 immunostaining, original magnification 40X). C. Groups of interstitial macrophages (CD68 immunostaining, original magnification 40X).

**Fig. 3:**
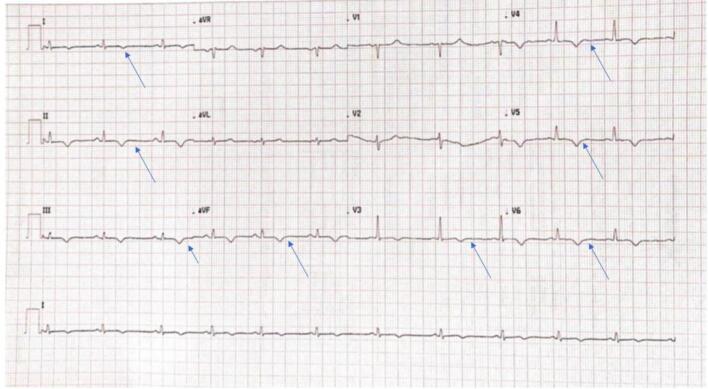
Twelve‑lead ECG at follow-up. Of note, low voltages were present in the peripheral leads, and negative T-waves were present from V3 -V6 leads.

Due to the clinical suspicion of AM, a CMR was performed revealing an extensive myocardial oedema with T2 sequences at the mid-basal segments of both lateral and inferior walls and at the level of the apex in toto, along with an increase in T1 and T2 mapping times at the same sites. The CMR confirmed the severe reduction of LVEF (25%) with a concomitant reduction of RVEF (46%), albeit of a lower degree (Fig. 1A). The imaging features were consistent with AM, but they did not indicate a specific underlying aetiology. Serological viral tests, including SARS-CoV-2, and autoimmune screens were negative. The patient did not improve despite the treatment. For this reason, an EMB was collegially indicated. Three fragments were taken from the LV. The histological examination revealed an increased number of interstitial CD68+ macrophages and CD3+ T lymphocytes (about 7 per mm^2^), focally organized in small clusters of 2–3 cells, without evidence of myocytes damage. There was mild interstitial oedema, with a modest increase of fibrous tissue, and focal myocardial fibro-adipose infiltration, limited to one of the myocardial fragments (Fig. 2). During the remaining hospitalisation the patient experienced a progressive clinical improvement, with a reduction of troponin level. She was gradually switched to an oral HF therapy. The subsequent ECHO documented a significant improvement of LVEF (45–50%). Lack of arrhythmias was documented by the ECG monitoring. At the follow-up visit, the ECHO showed a further improvement of ventricular function (LVEF 55%) and reduced mitral insufficiency. The ECG showed low voltages and negative T-waves in the infero-lateral leads (Fig. 3). A CMR performed after 4 months showed cardiac chambers of normal size and a full recovery of ventricular function (LVEF 65%, RVEF 57%). The subepicardial LGE was detected at the level of the mid-basal segments of the infero-lateral wall in the absence of oedema at T2 sequences and with normal T2 mapping time values (Fig. 1 B,C). Considering the clinical picture and the EMB result, a genetic analysis was collegially decided to investigate a possible underlying genetic predisposition.

The proband was referred for genetic counselling and analysis to the Medical Genetic Unit, Sant'Andrea Hospital, Rome. Genomic DNA was extracted from the peripheral blood using the DNeasy Blood & Tissue Kit and QIAamp DNA Blood Mini Kit (Qiagen, Hilden, Germany), according to the manufacturer's instructions. The genetic tests were performed after obtaining ethical approval and written informed consent of the proband. The genetic analysis was performed using a TruSight Cardio Panel (Illumina, San Diego, CA, USA) according to the manufacturer's instructions. The panel includes 174 genes related to cardiac diseases, and all core genes associated with ACM. The targeted regions underwent paired-end sequencing (2 × 150 bp cycles) on the MiniSeq platforms. For variant identification, we used a developed pipeline according to the Genome Analysis Toolkit (GATK) best practices for germline variant identification [9,10]. Raw data from NGS was aligned to the human genome reference GRCh37/hg19. The BaseSpace Variant Interpreter (Illumina, San Diego) was used for variant prioritization and annotation. Variants with read depth < 10×, quality <200, synonymous and intronic variants in non-splice regions and MAF higher than 0.01 were removed for further analysis. The identified variants were classified according to the ACMG criteria, to reports of both scientific literature and public databases (ClinVar, HGMD, OMIM, dbSNPs) [11]. The identified variant in *PKP2* was validated using NGS. The genotype analysis revealed a heterozygous variant in the *PKP2*. The frameshift variant NM_004572.3: c.148_151del, (p.Thr50Serfs*61), located on exon 1 of the *PKP2* gene, alters the plakophylin 2 protein at the 50 amino acid by introducing a premature termination codon with prediction of either an absent or truncated protein. According to ACMG criteria (PVS1, PM2), the variant was classified as likely pathogenic. The segregation study of the *PKP2* variant identified in the proband was performed in her two healthy sons. Both of them, free from cardiac disease history, resulted carriers of the variant. They were asymptomatic and without suggestive signs of ACM.

In addition to the *PKP2* variant, a VUS within the *ACTN2* was detected in the probamd: the missense variant c.1072C > T, located on exon 10 of the *ACTN2*, causing the substitution of the amino acid proline at position 358 by the amino acid serine in the protein actinin alpha 2. The variant is absent in the main general population consultation sites (GnomAD Genomes and Exomes), in the dbSNP, ClinVar, CardioClassifier, Atlas of Cardiac Genetic Variation databases, and in the scientific literature. The main prediction sites interrogated to evaluate the impact of the variant on the structure/function of the protein resulted in conflicting results. Therefore, according to ACMG criteria, the variant was classified as VUS.

## 3 Discussion and conclusions

The role of inflammation in ACM is not fully understood. In fact, it is unknown whether inflammation is a primary event leading to myocardial damage and arrhythmias or is a secondary response to cardiomyocytes death. Among other hypotheses, it has been proposed that an inflammatory, infective, or autoimmune trigger causes the onset and progression of the disease. Alternatively, the myocarditis-like episodes may represent an acute phase of ACM. Further research is needed to fully understand the molecular basis of the inflammatory process in ACM [2,12,13].

Clinically, patients may present with signs of AM, such as chest pain, elevated troponin level, ECG abnormalities, or myocarditis-like pattern on CMR.

A recent study performed in a cohort of 12 young females who presented with myocarditis syndrome (chest pain, troponin level elevation) reported that they were later diagnosed with ACM. These patients displayed distinct clinical and genetic characteristics, such as female predominance, LV involvement, and specific gene variants [2,14]. Lopez-Ayala et al. conducted an intriguing analysis on the incidence and genetic basis of AM in patients with ACM. They studied a population of patients with clinical and genetic diagnoses of either ARVC or ALVC (131 patients) and their genotype-positive family members (64 relatives). Over a median of 34 months, 6/131 (4.6%) patients, including one with ARVC, four with ALVC, and one with biventricular ACM, and 1/64 (1.6%) clinically healthy variant carriers developed acute chest pain, with associated elevated troponin level and progressive worsening of LVEF, without any episode of fever or infection in the weeks preceding AM. Interestingly, all patients with the AM-like episodes were carriers of damaging variants in *DSP* [8].

Recently, 336 patients with AM underwent targeted DNA sequencing for well-characterized cardiomyopathy-associated genes. As a result, genetic variants associated with DCM and ACM were identified in 8% of patients with AM. Notably, truncating variants of *DSP* were identified in 3% of cases versus 0.4% of controls (odds ratio, 8.2; *P* = 0.001), even in the absence of family history [2]. Previous genetic studies have implicated desmoplakin as the protein most frequently involved in ACM [7,8,12,15,16].

The frameshift variant LRG_3981t1: c.148_151del on *PKP2* identified in our proband is rare in major general population reference sites (GnomAD Genomes ƒ = 0.0000263). It is noted in dbSNP databases (rs397516997) ClinVar (Variant ID: 54195) as pathogenic and in Cardio Classifier as likely pathogenic (PM2) (PP5). This variant has been previously described in multiple individuals from unrelated families with ARVC, but also in healthy family members, suggesting an incomplete penetrance [17,18].

Functional studies demonstrated that this variant is responsible for reduced levels of mRNA and plakophilin 2 protein [19,20]. Null (nonsense, frameshift, splice) variants of *PKP2,* for which loss of function is a known mechanism of disease, are usually considered deleterious and are associated with autosomal dominant ARVD type 9 (MIM # 609040). The plakophilin 2 protein mediates the attachment to intracellular filaments and membrane proteins, anchoring desmoplakin and the intermediate filament to the desmosome structure.

The loss of function or haploinsufficiency of the plakophilin 2, or of other proteins involved in the desmosome plaque structure, may cause a structural and mechanical failure due to the instability of the DIFC [20]. Recently, it has been suggested that a plakophilin-2 mutation can result, through a ubiquitin-proteasome system-dependent mechanism, in reduced desmosome and adherent junction protein production. This evidence suggests a possible therapeutic approach for the management of ACM by the inhibition of protein targeting and degradation, and the enhancement of the desmosome protein stability [21].

Usually, carriers of null variants in *PKP2* have an earlier onset of ARVC, compared to carriers of missense mutations, with a risk of endpoint events (documented SVT episodes, ventricular fibrillation, SCD, etc) lower than *DSP* carriers [17,18]. *PKP2* variants carriers had also an earlier onset of symptoms and arrhythmias with a higher proportion of T wave inversion in V1–3 leads than subjects with negative genotypes [18,22]. Individuals carrying *PKP2*:c.148_151del may present with a variety of symptoms, including palpitations, syncope, chest pain, and HF. However, AM has never been associated with this variant. Other *PKP*2 pathogenic variants have been identified in patients with AM (either single or recurrent episodes) in the absence of a previous diagnosis of cardiomyopathy [23,24].

A peculiar aspect of our patients is that she had a fulminant AM with severe depression of LVEF and hypotension as the first manifestation of ACM. In the aforementioned studies, the majority of patients carrying mutations in the desmosomal genes had preserved EF during the myocarditis phase with district a/hypokinesias, although they had a higher arrhythmic risk and an extended LGE at CMR [25]. The onset with fulminant AM is more frequently seen in patients who subsequently develop DCM although the areas of LGE at CMR are less extensive [2]. The increased incidence of recurrent AM in ACM compared to the normal population is an important aspect because it emphasizes how AM characterizes not only the initial phase but also the entire course of ACM through the so-called ‘hot phase’ [15].

Our case report suggests that common diagnostic procedures such as ECG, Holter monitoring, echocardiography and CMR may be insufficient to distinguish between AM and ACM. The histological finding of a fibro-adipose replacement in the myocardium is certainly very important but, given the high rate of complications related to EMB, is not frequently performed. In these cases, the genetic analysis can provide straightforward insights. However, the genetic analysis cannot be performed in all patients due to its high cost and limited availability in most healthcare facilities. Therefore, the opportunity of genetic testing must be driven by specific features, called “red flags”, usually obtained from the above-mentioned diagnostic procedures. The most suggestive “red flags” reported in the literature are: the low voltages in the infero-lateral leads with T-wave inversion, an extensive late gadolinium enhancement at CMR (“ring-like pattern”), presence of ventricular arrhythmias, HF and recurrent AM [12,26,27]. Our patient had most of the “red flags” linked to ACM. This evidence prompted us to perform the genetic analysis that allowed a definitive diagnosis.

In conclusion, a clinically suspected AM can be the initial manifestation of a hereditary cardiomyopathy, particularly of ACM. The genetic background should be investigated in patients with suspected AM to identify factors that may affect disease progression and to perform a precise diagnosis of cardiomyopathy for a specific medical treatment and follow up.

## Ethics approval

This is an observational retrospective patient report that did not involve any research-based intervention. All interventions were intended to diagnose and treat the patient. The study was conducted in accordance with the Declaration of Helsinki, and the protocol was approved by the Ethics Committee of S. Andrea Hospital (approval identification number: 42 of 28 September 2017).

## Patient consent

Written informed consent for the present study was obtained from the patient's parents.

## Author contributions

RM, CM, FF, MF, SP: data curation and investigation; CS, AG, GDA, CG:: methodology and analysis; SR, MP, CA, AP, VV: supervision and writing. All authors approved the final manuscript as submitted and agreed to be accountable for all aspects of the work. All authors confirm the absence of previous similar or simultaneous publications.

## Declaration of Competing Interest

None.

## Data Availability

Data will be made available on request.
